# Assessment of Readiness for Discharge and Its Psychosocial Determinants in Kidney and Liver Transplant Patients in Poland—A Cross-Sectional Analytical Study

**DOI:** 10.3390/healthcare13233134

**Published:** 2025-12-02

**Authors:** Marta Katarzyna Hreńczuk, Patrycja Adamczyk, Dominika Niewierowska, Tomasz Dawid Piątek

**Affiliations:** Department of Surgical and Transplantation Nursing and Extracorporeal Therapies, Medical University of Warsaw, 02-006 Warszawa, Poland; patrycja.adamczyk@wum.edu.pl (P.A.); dominika.niewierowska@wum.edu.pl (D.N.); tomasz.piatek@wum.edu.pl (T.D.P.)

**Keywords:** kidney transplantation, liver transplantation, readiness for discharge, self-care, psychological resilience, depression, anxiety, social support

## Abstract

**Highlights:**

**What are the main findings?**

- Readiness for discharge after kidney and liver transplants mainly depends on patients’ self-care knowledge and their level of psychological resilience.
- Anxiety, depression, and social support were not significant predictors at discharge, although they might affect later stages of post-transplant recovery.
- The regression model confirmed the dominant role of cognitive-behavioral factors, while emotional and social variables showed only minimal effect sizes.

**What is the significance of the main finding?**

- Self-care education and brief resilience-building interventions should become standard parts of discharge planning in transplant care.
- Enhancing cognitive and behavioral skills may lower the risk of rehospitalization, improve adherence, and promote better long-term post-transplant results.
- Clinical practice should include structured discharge-readiness assessment tools that encompass both clinical and psychosocial resources.

**Abstract:**

**Background/Objectives**: Readiness for hospital discharge after kidney and liver transplantation is a complex process influenced not only by clinical factors but also by psychological and social aspects. This cross-sectional, analytical study aimed to assess discharge readiness and examine the psychological and social factors affecting patients’ preparations for leaving the hospital among renal and liver transplant recipients. **Methods**: A diagnostic survey was conducted using standardized questionnaires, including assessment of readiness for discharge (based on Canadian Health Outcomes for Better Information and Care-C-HOBIC), Hospital Anxiety and Depression Scale (HADS), Multidimensional Scale of Perceived Social Support (MSPSS), and Resilience Measurement Scale (SPP-25). The collected data were analyzed using IBM SPSS Statistics version 28. The study included 117 recipients (31.6% liver, 68.4% kidney) who received transplants at centers in Poland between February and May 2025. **Results**: The average discharge readiness score was 34.51 ± 5.37. Overall, 79.5% of respondents had high discharge readiness, while 20.5% reported moderate readiness. A multiple regression model with eight predictors (SPP-25, HADS-depression, HADS-anxiety, Friends, Family, Significant person, Knowledge of self-care, Communication and information support) was statistically significant (F(8,108) = 11.629, *p* < 0.001), explaining 46.3% of the variance in discharge readiness (R^2^ = 0.463; adjusted R^2^ = 0.423). Knowledge of self-care emerged as the strongest predictor (B = 0.475, β = 0.577, *t* = 5.424, *p* < 0.001), followed by psychological resilience (SPP-25) (B = 0.011, β = 0.221, *t* = 2.381, *p* = 0.019). Other variables were not significant predictors (*p* > 0.05). **Conclusions**: Discharge readiness after kidney and liver transplantation is significantly affected by patients’ self-care knowledge and psychological resilience, highlighting the importance of education and psychological support in preparing patients for discharge.

## 1. Introduction

Transplantation is now the standard treatment for terminal organ failure, such as liver or kidney failure. It not only significantly prolongs life but also considerably improves quality of life compared to conservative treatments like dialysis in renal failure [1,2,3,4]. In recent decades, there has been steady progress in recipient survival, driven by advances in surgical techniques, improvements in immunosuppression methods, and better donor and recipient selection criteria [4,5].

According to the Global Observatory on Donation and Transplantation (GODT), over 170,000 organ transplants were performed worldwide in 2023, marking a significant increase compared to previous years [6]. There is also a noticeable upward trend in transplant numbers in Europe [7]. The situation in Poland mirrors both global and regional patterns [8].

Health systems face pressure to use resources efficiently, including reducing hospital stay durations. In clinical practice, the decision to discharge a patient after transplantation must balance the need to free up hospital beds quickly with ensuring patient safety and minimizing risks such as rehospitalization, organ rejection, or infectious complications. Additionally, the changing demographics of recipients—who are increasingly older or have multiple health conditions—further complicate discharge planning [5]. Longer post-transplant survival and the requirement for long-term immunosuppressive therapy mean that patient care demands thorough monitoring, support, and education, which should start even before the transplant and continue during hospitalization [1,4]. Equally important is the active involvement of the recipient in their treatment, especially practicing self-care during the post-hospital period. All these factors emphasize the critical need for research on discharge readiness among transplant patients.

Discharge from hospital after a kidney or liver transplant is a crucial step in the entire treatment process, marking the shift from intensive, specialized medical monitoring to the patient’s independence at home. Its significance goes beyond logistics—the discharge decision reflects an evaluation of the patient’s clinical stability and readiness to manage self-care, as well as the availability of support in the community [9,10].

Safe discharge offers several benefits: shorter hospital stays, lower risk of hospital-related complications (e.g., nosocomial infections), reduced healthcare costs, and an increased sense of empowerment and quality of life for patients [11,12]. However, discharging patients too early or without proper preparation can raise the risk of rehospitalization, worsen clinical outcomes, and cause emotional destabilization for patients and their families [13,14,15].

In transplantology, the timing of discharge is especially important because these patients need to follow medical recommendations carefully every day. This includes taking immunosuppressive medication at the right doses and times, monitoring health parameters (blood pressure, blood sugar, body weight, signs of infection or transplant rejection), and undergoing regular laboratory tests [16,17]. Therefore, the patient’s readiness to meet these requirements becomes a crucial safety factor when deciding to end hospitalization.

Research indicates that assessing readiness for discharge should encompass not only clinical factors but also psychosocial aspects—including the patient’s understanding of recommendations, anxiety and depression levels, access to family and caregiver support, and system resources like the ability to contact the medical team after leaving the hospital [18]. A well-organized process of educating the patient and their support network, along with formal tools such as questionnaires to evaluate readiness for discharge, can greatly reduce the risk of unplanned rehospitalizations and enhance long-term outcomes after transplantation [19,20].

Readiness for hospital discharge (RHD) is a concept that includes both objective clinical criteria and the patient’s subjective feelings about feeling safe to return home. The literature emphasizes that this assessment is multidimensional and that reliable evaluation is crucial for health outcomes, such as reducing the risk of rehospitalization and complications [19,20,21].

Weiss et al. define RHD as how much the patient and their family feel ready to transition from hospital care to self-care at home [22]. This includes several key aspects, such as: clinical status (like stability of vital signs, lab results, and function of the transplanted organ); patient knowledge and skills (such as understanding treatment recommendations, pharmacotherapy—like immunosuppressive drugs—diet, and self-care); self-care ability (the level of independence in doing daily activities and following medical advice); emotional state (such as anxiety, depression, sense of competence, and sense of control); and social and environmental support (availability of family, caregivers, and system resources, e.g., easy access to the transplant center, and the ability for quick intervention if problems arise) [9,10]. Classical definitions by Weiss et al. [22] assume that readiness for discharge includes these components: emotional state, sense of control and competence, self-care knowledge, and social support. However, for organ transplant recipients, this concept needs to be expanded to cover factors related to long-term adjustment to living with a transplanted organ.

In recent years, there has been increased focus on cognitive–behavioral mechanisms of health regulation, highlighting that traits such as psychological resilience are crucial for adaptive processes. Research confirms that greater resilience enhances effective coping with perioperative stress and improves the ability to absorb educational content, which subsequently leads to higher readiness for independent functioning after the procedure [23]. Additionally, a systematic review of interventions among kidney transplant recipients showed that health education and self-management programs significantly influence cognitive, behavioral, and emotional outcomes, underscoring the importance of education in preparing patients for independent living after discharge [24]. Similarly, in liver transplant recipients, empowerment-based educational interventions have been found to significantly boost self-care and self-efficacy, aiding better post-transplant adaptation [25]. Despite the increasing number of studies emphasizing the importance of education and psychological resilience, few analyses have explored their interrelationships among organ transplant recipients—especially within the Polish healthcare setting—and the influence of social support, anxiety, and depression on these relationships. These findings support the theoretical framework underlying the core elements of discharge readiness. The model aligns with Meleis’ Transition Theory, which suggests that successful progression through a health-related transition depends on sufficient knowledge and preparation, personal resources like psychological resilience, and appropriate social and environmental support [26].

Integrating these three domains places the variables studied (resilience, anxiety, depression, social support, and self-care knowledge) within a coherent theoretical framework that aligns with current trends in nursing and transplantology.

In the case of kidney and liver transplant recipients, evaluating readiness for discharge and influencing factors is especially important, as these patients need immediate and consistent adherence to medical advice. Studies indicate that low discharge readiness is linked to a higher risk of non-adherence, poorer post-transplant adaptation, and more frequent rehospitalizations [15,27].

Transplantology in Poland operates within a centralized system managed by Poltransplant and includes a network of specialized clinical centers offering comprehensive inpatient and outpatient post-transplant care. In practice, transplant coordinators and ward staff play a key educational role, while post-discharge care is provided through outpatient transplant clinics, including follow-up visits and monitoring of immunosuppressive therapy. However, structured, multi-stage transitional care programs are not yet established in Poland, and studies on the psychosocial factors affecting discharge readiness are almost non-existent.

Although international evidence demonstrates the effectiveness of educational and transitional care interventions in improving post-transplant outcomes—for example, the randomized controlled trial by Hu et al. [20], which showed that a structured transitional care program enhanced discharge readiness, care quality, and patient satisfaction among kidney transplant recipients in China—there remains a lack of research conducted within the context of the Polish healthcare system. Specifically, no studies have assessed how individual psychosocial factors, such as self-care knowledge, psychological resilience, social support, anxiety, and depression, influence patients’ readiness for hospital discharge after organ transplantation.

A readiness assessment can help identify patients who may need additional support or intervention to ensure a successful discharge. The reviewed literature shows a lack of comprehensive research on this patient group. Therefore, the aim of the study was to evaluate the readiness for discharge of kidney and liver transplant patients, identify its psychosocial and psychological determinants, and attempt to find its key predictors, which may aid in planning targeted educational activities.

The study is the initial phase of presenting results and does not evaluate clinical measures of readiness or examine events such as early hospital readmissions, frequency of reporting to outpatient clinics or emergency departments, functioning of the transplanted organ, or quality of life at a specific time after hospital discharge.

## 2. Materials and Methods

### 2.1. Study Design

The study was carried out at four university-affiliated clinical hospitals in Poland, all of which are accredited and authorized to perform transplant surgeries. One of the centers included three transplant wards, two other centers had one each, and the last had two. Among these facilities, three performed liver transplants, five performed kidney transplants, and one center performed both procedures (kidney and liver). The remaining hospitals specialized solely in transplanting a single organ. All participating centers were comparable in terms of educational and welfare infrastructure, as well as medical and nursing care standards, since they operate within a centralized national transplantation system and follow uniform nationwide quality and accreditation standards.

### 2.2. Participants

The study was a cross-sectional analytical investigation involving 117 organ recipients (31.6% liver recipients and 68.4% kidney recipients) hospitalized in Polish transplant centers between February and May 2025, with the centers’ managers consenting to participate. This study did not perform a formal sample size calculation, which aligns with recommended practices for exploratory studies of clinical populations with limited accessibility. According to methodological guidelines for cross-sectional studies in special populations (e.g., organ transplant recipients), using a “consecutive sampling” strategy is acceptable, especially when the population is small, difficult to access, and clinically diverse. In such cases, the basis for considering a sample sufficient is not a formal statistical formula, but rather meeting two key criteria: adequate variability saturation, enabling stable statistical estimates, and adherence to the recommended sample size for multivariate analyses of 5–10 observations per analytical variable.

In this study, both criteria were met. Out of the 150 questionnaires distributed, 117 completed ones were collected, representing about one-sixth of all kidney and liver transplant recipients hospitalized during the studied period. The sample size exceeds the minimum values suggested in the methodological literature for regression models, correlation analyses, and validation studies [28,29]. Importantly, studies examining discharge readiness and psychosocial functioning in organ transplant recipients often rely on comparable or smaller samples—e.g., 80–120 participants—confirming that the obtained sample size meets research standards in this field.

Using total population sampling maximized representativeness within the specific clinical group and minimized the risk of selection bias. Due to the sparse population, clinical diversity, and exploratory nature of the study, this data collection approach is considered methodologically appropriate and aligns with current standards for descriptive and explanatory clinical research.

Inclusion criteria: patients who have undergone kidney or liver transplantation, the ability to complete the survey independently, age 18 or older, and consent to participate in the study.

### 2.3. Data Collection

The questionnaires were completed in a hospital setting on the day before discharge, in the presence of a member of the research team or a transplant coordinator. Participants had the option to complete the questionnaire either on paper or electronically (via QR code). Most respondents (n = 111) chose the paper version, while six participants selected the online format. Data from both sources were standardized through a consistent coding and data entry process using IBM SPSS Statistics software (v. 28). Quality control procedures (double-entry verification) confirmed there was no data loss or inconsistencies between the two versions of the instrument.

### 2.4. Instruments

A diagnostic survey method was employed using 4 questionnaires that are standardized and validated for Polish conditions.

− The Hospital Discharge Readiness Assessment Questionnaire is a self-report tool from the Canadian Health Outcomes for Better Information and Care (C-HOBIC) project. Through adaptation, the tool achieved internal consistency reliability, with a Cronbach’s alpha of 0.816. Its purpose is to evaluate self-care readiness. The questionnaire includes 8 questions covering: knowledge of medicines, understanding the reasons for taking medicines, ability to take prescribed medications, recognizing health-related symptoms, adhering to treatment, knowing whom to contact for help with daily activities, knowing whom or which institutions to call in case of emergency, and the ability to engage in daily physical activity. Each question offers 6 response options for participants to choose from: 0—not prepared, 1—poorly prepared, 2—partially prepared, 3—moderately prepared, 4—well prepared, 5—very well prepared. The total discharge readiness score ranges from 0 to 40 points, with higher scores indicating better discharge readiness. The discharge readiness scale derived from the C-HOBIC set has established content and construct validity in populations of chronically ill and post-surgical patients. Validation studies demonstrated high internal consistency, with Cronbach’s alpha ranging from 0.86 to 0.91 [30,31];
− The Hospital Anxiety and Depression Scale (HADS) includes 14 items divided into two subscales, each assessed separately: 7 items measure anxiety—HADS-Anxiety (HADS-A)—and 7 items evaluate depression—HADS-Depression (HADS-D). The individual taking the test indicates each symptom by selecting one of four options that best describe how they have been feeling over the past week. Responses are scored on a 4-point Likert scale (from 0 to 3 points). Total scores range from 0 to 21, with higher scores indicating greater severity of symptoms. A score of 0–7 is considered normal, 8–10 is borderline and suggests a possible disorder, while 11 or higher is deemed pathological, indicating a probable presence of a disorder (for both anxiety and depression). In its Polish edition, HADS demonstrates strong psychometric properties, with a Cronbach’s alpha of 0.83 for the anxiety subscale and 0.82 for the depression subscale. Its validity has been established through both population-based and clinical research, confirming it as a reliable screening tool for affective symptoms [32,33];
− The Multidimensional Scale of Perceived Social Support (MSPSS) examines different aspects of perceived social support by focusing on three main sources: significant other, family, and friends. Respondents rated their agreement on a 7-point Likert scale from 0 (strongly disagree) to 7 (strongly agree). Higher scores indicate a stronger sense of social support and a greater belief in the availability of emotional, instrumental, and informational help. This scale is commonly used in both clinical and population studies, helping to assess how social relationships affect recovery, adaptation to chronic illness, and psychological functioning. The Polish version of the MSPSS demonstrates excellent reliability (Cronbach’s α = 0.93 for the total scale; 0.90–0.92 for the subscales: Family, Friends, and Significant Other). Construct validity has been confirmed in numerous studies evaluating perceived social support among both chronically ill and healthy groups [34];
− The Psychological Resilience Scale (SPP-25) assesses patients’ psychological resilience, which is their capacity to cope with difficult situations. It consists of 25 statements related to resilience across various scenarios. A higher score signifies greater resilience. Responses are rated on a five-point Likert scale from 0 (definitely no) to 4 (definitely yes). The results are categorized into five groups, each with five questions: perseverance and determination in action, openness to new experiences and humor, personal competence in coping and tolerating negative emotions, tolerance of failure and viewing life as a challenge, and an optimistic attitude with the ability to mobilize during tough times. The total SPP-25 score can be converted to a sten scale. Developed by Ogińska-Bulik and Juczyński, the SPP-25 demonstrates high reliability, with a Cronbach’s alpha of 0.89 for the overall scale and subscale values ranging from 0.67 to 0.75. Its validity is supported by positive correlations with measures of sense of coherence and life satisfaction [35].

The questionnaire was supplemented with metric questions that allowed the study group to be characterized in terms of sociodemographic and clinical data. Questions were also formulated concerning the assessment of communication and information support during the hospital stay and knowledge of self-care. Additionally, a subjective assessment of readiness for discharge was included, with responses given on a five-point Likert scale from 1 strongly disagree to 5 strongly agree.

### 2.5. Statistical Analysis

The collected material was analyzed using the IBM SPSS Statistics package (v. 28). Quantitative variables were summarized by mean, standard deviation, median, and range (minimum and maximum). For qualitative variables, the frequency and percentage of each category are presented. The two groups were compared with the Mann–Whitney U test. A Kruskal–Wallis rank-sum ANOVA was used to analyze more than two independent groups. Spearman’s rank correlation coefficient was applied to assess the relationship between variables.

The relationship between the dependent variable and the set of predictors was analyzed using multiple linear regression. The model included the following independent variables: communication and information support, relationship with a significant other, level of depression (HADS-depression), support from friends and family, knowledge of self-care, level of anxiety (HADS-anxiety), and indicators of self-care and social support (SPP-25). Model fit was evaluated using the multiple correlation coefficient (R), the coefficient of determination (R^2^), and the adjusted R^2^, which accounts for the number of predictors and sample size. Additionally, the standard error of the estimate was calculated as a measure of the average deviation of observed values from those predicted by the model. The results of the analyses were considered statistically significant at *p* < 0.05.

During the paper’s preparation, the authors used ChatGPT-5 to develop Tables and help interpret the data. After using this tool, the authors reviewed and revised the content and take full responsibility for the published work.

### 2.6. Ethics

The study was conducted in accordance with the Declaration of Helsinki. The Local Bioethics Committee took note of the information on the study, indicating that it complied with the principles of research ethics (AKBE/7/2025). Participation in the study was voluntary and anonymous. The completed questionnaires were placed by the respondent in an anonymous envelope and sealed, and were collected by a member of the research team.

## 3. Results

The 117 subjects were mostly male (56.4%), with a mean age of 47.64 ± 13.57 years. Nearly half of the respondents lived in rural areas (41%), while the rest resided in urban areas. Secondary education (45.3%) and tertiary education (36.8%) were most common. More than half of the respondents were employed (56.4%). Patients who had undergone kidney transplantation predominated (68.4%), and for 87.2% of them, it was their first transplant. The average length of hospital stay was 19.62 ± 11.49 days, and 35% experienced prolonged stays due to complications (Table 1).

Professional pre-transplant education was received by 76.1% of respondents, with 67.5% undertaking educational activities on their own, mainly by searching for information on the Internet (91.1%). Post-transplant education included 82.1% of respondents, with 68.4% receiving educational materials during their hospital stay (Table 2).

The overall discharge readiness score averaged 34.51 ± 5.37. Ninety-nine percent of respondents showed a high level of readiness for discharge, while 20.5% reported moderate readiness. Participants in the study demonstrated high levels of knowledge and competence related to medication and health management. Notably, the highest averages were in the ability to accept assigned medications and follow treatment recommendations, whereas the lowest scores were in noticing health changes and knowing whom to turn to for help with daily activities (Table 3).

Subjective readiness and self-care knowledge were rated above 4. Communication and information support received high ratings. The distribution of this variable showed strong negative skewness (A = −2.09) and high kurtosis (K = 5.31), indicating a significant skew toward high scores in this area (Table 4).

A comparative analysis of results based on sex, education level, and place of residence showed no statistically significant differences in readiness for discharge (*p* > 0.05). The age of respondents had a weak, mostly statistically insignificant relationship with the assessment of readiness for discharge and its specific aspects. Significant negative correlations were only found in two areas: knowledge about the reasons for taking medication and the ability to follow treatment recommendations. This suggests that understanding of pharmacotherapy reasons and the ability to consistently adhere to medical advice may slightly decline with age. Regarding the occupational status of subjects, a statistically significant difference was observed in the ability to take prescribed medication, favoring employed respondents, who demonstrated a higher level of this ability than unemployed respondents. Additionally, there was a significant difference in knowledge about contacts for help with daily activities—allied with a higher level of knowledge among employed respondents. No statistically significant differences were found between groups in the other variables analyzed (Table 5).

Correlation analysis between the length of hospital stay, type of transplanted organ, prolongation of hospitalization due to complications, and overall assessment of readiness for discharge along with its individual aspects showed no statistically significant correlations (*p* > 0.05). In an analysis comparing first-time transplant patients with those who had subsequent transplants, a statistically significant difference was only found in knowledge about institutions or persons to contact in case of an emergency. Patients with a subsequent transplant demonstrated higher levels of this knowledge compared to first-time transplant patients (Table 6).

Correlation analysis between the overall readiness for discharge and the components of psychological resilience (SPP-25) revealed statistically significant, positive relationships with all examined dimensions. The strongest correlations were seen for an optimistic outlook on life and the ability to mobilize in challenging situations (rho = 0.428; *p* < 0.001), as well as tolerance for failure and viewing life as a challenge (rho = 0.406; *p* < 0.001). The total SPP-25 score was also strongly linked to readiness for discharge (rho = 0.389; *p* < 0.001). Slightly weaker but still significant correlations appeared for openness to new experiences and a sense of humor (rho = 0.345; *p* < 0.001), personal competence and tolerance for negative emotions (rho = 0.338; *p* < 0.001), and perseverance and determination to act (rho = 0.253; *p* = 0.006). These findings suggest that higher levels of psychological resilience are associated with better patient preparedness for hospital discharge.

Positive associations were observed between overall discharge readiness and individual sources of social support: friends (rho = 0.147; *p* = 0.113), family (rho = 0.131; *p* = 0.159), and significant others (rho = 0.166; *p* = 0.073). The total MSPSS scale score displayed a low correlation (rho = 0.144; *p* = 0.122). None of these associations reached statistical significance (*p* > 0.05).

Correlations showed weak, negative links between overall discharge readiness and levels of depressive symptoms (rho = −0.204; *p* = 0.028) and anxiety (rho = −0.182; *p* = 0.050) as measured by the HADS scale. The depression score was statistically significant, while the correlation with anxiety was borderline significant. This indicates that higher levels of depressive and anxiety symptoms are connected to lower ratings of discharge readiness.

Correlations between subjective readiness for discharge and individual dimensions of psychological resilience showed only very weak positive correlations that did not reach statistical significance. In contrast, correlations between subjective readiness for discharge and social support were weakly positive. The strongest association was found with the combined score of the MSPSS scale (rho = 0.202; *p* = 0.029) and family support (rho = 0.200; *p* = 0.030). Support from a significant other also showed a significant, but slightly weaker, correlation (rho = 0.182; *p* = 0.049). Correlations between subjective readiness for discharge and psychopathological symptoms were weak, negative, and not statistically significant.

Correlations between self-care knowledge levels and aspects of psychological resilience showed moderate, positive, and partly statistically significant relationships. The strongest links were found for tolerance of failure and viewing life as a challenge (rho = 0.306; *p* = 0.001) and for maintaining an optimistic attitude and the ability to mobilize during difficult times (rho = 0.291; *p* = 0.001). Significant correlations also appeared for personal competence in managing and tolerating negative emotions (rho = 0.238; *p* = 0.010) and for openness to new experiences coupled with a sense of humor (rho = 0.212; *p* = 0.022). The overall SPP-25 scale score also showed a significant, moderate correlation with self-care knowledge (rho = 0.271; *p* = 0.003). However, correlations between self-care knowledge and social support were weak and positive, not reaching statistical significance. Anxiety symptoms (HADS—anxiety) were significantly, though weakly, negatively associated with self-care knowledge (rho = −0.214; *p* = 0.021).

The communication and information support received by respondents showed a positive, though moderate, correlation with openness to new experiences and a sense of humor (rho = 0.261; *p* = 0.004), personal competence in coping and tolerance of negative emotions (rho = 0.261; *p* = 0.004), and tolerance of failure and viewing life as a challenge (rho = 0.270; *p* = 0.003). The highest correlation was with an optimistic outlook on life and the ability to mobilize in difficult situations (rho = 0.345; *p* < 0.001). Additionally, communication and information support were significantly linked to the overall SPP-25 scale score (rho = 0.291; *p* = 0.001). They also correlated with support from friends (rho = 0.331; *p* < 0.001) and general support measured by the MSPSS scale (rho = 0.313; *p* = 0.001). Significant correlations were found with family support (rho = 0.291; *p* = 0.001) and support from a significant other (rho = 0.229; *p* = 0.013). Negative correlations between communication and information support and levels of depressive symptoms (rho = −0.194; *p* = 0.036) and anxiety (rho = −0.317; *p* < 0.001), as measured by the HADS scale, were observed.

The correlations between general readiness for discharge, subjective readiness for discharge, knowledge about self-care, communication and information support, and the components of mental resilience, social support, and levels of depression and anxiety symptoms are presented in Table 7.

The results of the correlations between subjective readiness, communication and information support, and knowledge of self-care and various aspects of readiness for hospital discharge were found to be significant and positive. The strongest correlations are between knowledge of self-care and the overall assessment of readiness for discharge (rho = 0.660; *p* < 0.001), and between communication and information support and the same aspect (rho = 0.611; *p* < 0.001). Similarly, all variables analyzed, such as knowledge of medication, ability to take medication, or ability to notice health symptoms, show significant positive correlations with three factors: subjective readiness, communication and information support, and knowledge of self-care (all *p* < 0.05) (Table 8).

The regression model with eight predictors (SPP—25, HADS-depression, HADS-anxiety, Friends, Family, Significant person, Knowledge of self-care, Communication and information support) was found to be statistically significant in explaining variation in readiness for discharge (F = 11.629, *p* < 0.001). The regression sum of squares was 24.218, and the residuals (error) was 28.114, indicating that the model explains a significant portion of the variance in the dependent variable. The multiple correlation coefficient R = 0.680 suggests a meaningful positive relationship between the set of predictors and the outcome variable. An R-square of 0.463 shows that approximately 46.3% of the variance in the dependent variable is explained by the model, while an adjusted R-square of 0.423, which accounts for the number of predictors and sample size, confirms that the model is a relatively good fit without overfitting. The standard error of estimate, 0.51021, indicates that the model’s predicted values are moderately accurate and deviations from observed values are relatively small.

The results showed that the SPP-25 was a significant predictor, with higher pressure levels linked to a small but statistically significant increase in the dependent variable (B = 0.011, β = 0.221, *t* = 2.381, *p* = 0.019). While statistically significant, the standardized effect size (β = 0.221) suggests a modest practical impact, indicating that resilience plays a meaningful but not strong role in discharge readiness. In contrast, knowledge of self-care emerged as the strongest factor (B = 0.475, β = 0.577, *t* = 5.424, *p* < 0.001), showing that individuals with better understanding of self-care strategies scored higher on the dependent variable. The effect size for this predictor (β = 0.577) demonstrates a substantial and practically meaningful influence, making it the main cognitive-behavioral factor in the model.

The other variables analyzed, such as symptoms of depression and anxiety (HADS), social support from friends, family, and a significant other, and communication and information support, showed no significant effect (all *p* > 0.05). Despite their theoretical relevance, the lack of statistical significance and very small effect sizes (all β < 0.05) suggest that their explanatory power in this model is limited. This distinction highlights that some psychosocial factors, while conceptually relevant, contribute minimally to the variance in discharge readiness when evaluated alongside stronger predictors. VIF values were below the critical level of 5, indicating no significant collinearity between predictors (Table 9).

## 4. Discussion

The study confirms that assessing readiness for discharge after kidney and liver transplants should not rely solely on clinical parameters but requires a holistic, patient-centered approach. By design, study patients were discharged with stable laboratory and clinical parameters, which were not analyzed in this study. The indicators chosen were based on the definitions of readiness for discharge provided by Weiss et al. [22], aligning with key aspects of transition theory [36] and covering educational, psychological, and social domains.

The study’s participants were characterized by a high readiness for discharge, both according to the C-HOBIC assessment and their self-assessments. They rated their ability to take medication and follow treatment recommendations as excellent, which plays an important role in self-care. Conversely, they were least prepared to notice changes and, to their knowledge, support options available. Most sociodemographic data did not correlate with patients’ discharge readiness; however, it was observed that understanding the reasons for pharmacotherapy and the ability to follow recommendations may decline with age. This is an important finding, given the increasing number of transplants among the elderly. These individuals (≥65 years) are the fastest-growing age group undergoing transplantation [37,38,39]. For example, among kidney recipients, the number of elderly patients has tripled over the past decade, now accounting for nearly one-fifth of the population [40,41]. Other studies have noted that prolonged hospitalization and complications such as rejection significantly reduce discharge readiness [42]. However, in this study, prolonged hospitalization due to complications was not significant. The work situation and previous transplantation experience influenced preparedness only in a few areas.

The results suggest that high readiness for discharge is associated with lower anxiety and depression levels and greater psychological resilience. While stable support and its sources did not correlate with the results of the C-HOBIC questionnaire, they were significant in respondents’ self-assessments of readiness. Those who received support from family and significant others perceived their discharge readiness more positively. These findings are consistent with other studies emphasizing that meeting clinical criteria alone does not guarantee a safe discharge; emotional and environmental factors are also crucial [13,22,43]. Similar observations in the literature highlight the importance of psychological support and reduced anxiety and depression levels, indicating that psychosocial factors influence clinical outcomes such as survival, quality of life, and adherence [44,45]. The multiple regression analysis further confirms these relationships, demonstrating that discharge readiness after transplantation is multifaceted, involving both personal traits and educational components. The model was statistically significant, accounting for 46.3% of the variance in discharge readiness. Among the predictors, knowledge of self-care was the strongest; patients who were better informed about coping strategies post-discharge felt more prepared. This aligns with previous research showing that education and preparation for self-care are essential for high discharge readiness and fewer rehospitalizations [46]. Other studies have also confirmed that self-care knowledge is a key predictor of discharge readiness, showing that educational programs and self-management interventions improve self-care skills and clinical outcomes while enhancing cognitive and behavioral competencies [23,47]. Notably, the effect size for self-care knowledge (β = 0.577) indicates a significant and clinically meaningful contribution, supporting its role as a central cognitive-behavioral factor in the discharge process. The second significant predictor was mental toughness, which, despite a moderate impact, remained statistically significant. However, its standardized effect (β = 0.221) suggests only a modest practical influence, implying that resilience contributes meaningfully but does not dominate the explanatory structure of the model. Similar findings in resilience research show that higher resilience levels help individuals better cope with chronic illness and adapt to new health conditions [48,49,50]. Overall, these results suggest that educational programs designed to develop self-control and self-care skills in organ transplant recipients positively influence their cognitive, behavioral, emotional, and health functions. Those with higher resilience can better manage anxiety and stress, aiding in the adoption and reinforcement of health-promoting behaviors and boosting self-efficacy. Consequently, this may improve social adaptation and enhance both physical and psychological well-being among transplant recipients [51].

The lack of significant effects for anxiety, depression, and social support levels may indicate that, at discharge, these factors are not directly linked to feelings of readiness but might have a larger role during later stages of adaptation and recovery after transplantation. Their very small effect sizes in the model (all β < 0.05) further suggest that their immediate explanatory power is limited, despite their theoretical importance. These findings imply that, during discharge, practical elements like knowledge and self- care skills, along with individual resources such as psychological resilience, are prioritized, while emotional and social factors may influence long- term recovery and adjustment to life with a transplanted organ. Several studies show that the impact of anxiety and depression on post- transplant outcomes becomes evident mainly between 3 and 6 months after hospital discharge, when patients begin functioning independently at home [52,53,54,55]. An analysis by Guo et al. [30] indicated that social support has an indirect effect by strengthening education and psychological resilience but does not directly predict discharge readiness. Other research suggests that the perception of social support in post- transplant populations may be “saturated,” since most patients receive constant family and systemic care. As a result, the variability of this factor is limited, making it difficult to detect a statistically significant effect [56]. Overall, these results support the idea that discharge readiness is primarily shaped by cognitive- behavioral factors, while emotional and social variables may have indirect or delayed effects in the post- transplant process. From an international perspective, similar patterns have been observed in East Asian and US transplant settings, where structured educational programs and empowerment- based interventions strongly influence discharge readiness despite notable cultural and system-level differences [20,25]. Conversely, studies from high- income countries with more varied discharge practices suggest that social support and psychosocial burden play a more substantial role for certain patient groups, especially among minorities or those managing fragmented care systems [57]. In more uniform, publicly funded systems such as those in Central and Eastern Europe, the perceived saturation of social support might limit its variability and reduce its predictive effect [58,59]. These cross- country differences highlight that cognitive- behavioral factors appear consistent across health systems, while the influence of social and emotional factors may vary depending on cultural norms and healthcare organization.

Our findings highlight the dominant role of cognitive-behavioral resources (resilience, knowledge), which complements traditional models that mainly focus on the patient’s clinical condition. This difference emphasizes the practical importance of enhancing patients’ self-management skills at discharge, even when other psychosocial factors have minimal immediate impact.

### 4.1. Implications for Practice

The results of the study have important clinical implications for the care of kidney and liver transplant patients, particularly in planning for safe hospital discharge. First, they highlight that assessing readiness for discharge should include not only clinical parameters and stabilization of the physical condition but also measuring the patient’s level of self-care knowledge and psychological resilience. This emphasizes that patient education and preparation during hospitalization are crucial parts of the discharge process. Additionally, the results underscore the importance of educating both the patient and their support network so they have the necessary knowledge and skills for self-care. Education should be an ongoing process, starting from the initial days of hospitalization, covering topics such as immunosuppressive treatment, diet, self-management, and the development of emotional skills and empowerment. Other studies indicate that well-planned education reduces the risk of non-adherence and rehospitalization [48,60]. The fact that psychological resilience was a key predictor suggests the need for psychological interventions to build resilience, such as short-term psycho-educational programs or support from a clinical psychologist in the transplant unit. Furthermore, employing specialized tools that combine psychological assessment with therapeutic education could serve as a basis for creating personalized post-transplant rehabilitation models in routine clinical practice. To enhance practical application, resilience-building and educational programs can be designed as structured, evidence-based interventions. For example, resilience training may involve brief cognitive-behavioral sessions (30–45 min) focusing on stress management, cognitive reframing, and strengthening coping strategies, delivered 2–3 times during hospitalization [61]. These interventions can be carried out by clinical psychologists or trained nurse educators and monitored using validated scales to track progress over time.

Similarly, educational programs should be modular, using checklists and teach-back methods, covering key areas of self-care (medication management, symptom monitoring, infection prevention, lifestyle, and diet) [20,25]. Their effectiveness can be measured through outcomes such as improvements in knowledge tests, decreases in post-discharge phone consultations, adherence rates, or 30-day readmission rates. This approach would support personalized discharge preparation, allowing interventions to be tailored to each patient’s individual needs, resources, and self-care abilities. Incorporating these structured components into routine practice may ensure that discharge readiness becomes not only an assessment but also a goal for systematic, measurable clinical intervention.

### 4.2. Limitations of the Study

Limitations of the study include its cross-sectional design and absence of long-term follow-up. We cannot definitively determine how subjective readiness for discharge correlates with actual clinical outcomes, such as rehospitalization rates, adherence to treatment, function of the transplanted organ, or patient survival. Additionally, the literature indicates that low levels of perceived readiness for discharge may predict poorer clinical and psychological outcomes in subsequent treatment [17]. Another limitation is the reliance on self-report tools, which introduces the risk of response bias. The patient’s self-assessment may be influenced by temporary factors like mood, fatigue, or recent hospitalization experiences. Future research should include prospective and intervention studies to assess the effectiveness of educational and psychological programs aimed at enhancing patients’ readiness for discharge. Furthermore, the study sample was limited to patients from only a few transplant centers, restricting the generalizability of the results to all centers within the country and Europe. Variations in clinical practices, length of hospitalization, and availability of community support can impact discharge readiness levels and lead to different outcomes.

In conclusion, the study demonstrates that readiness for discharge after kidney and liver transplantation is a complex, multidimensional process, with self-care knowledge and psychological resilience being the key predictors. These results emphasize the importance for transplant centers to adopt care models that incorporate patient education, psychological evaluation, and psychosocial support into the routine discharge procedure.

## 5. Conclusions

The study results highlight the need to implement integrated and standardized discharge preparation programs that combine therapeutic education, psychological support, and systematic assessment of patient readiness, delivered by interdisciplinary teams of specialists. Specifically, it is recommended that these programs be structured to include modular self-care training along with brief interventions aimed at enhancing psychological resilience, with their effectiveness monitored using standardized tools. The successful implementation of these activities may also be aided by digital tools that support patient self-education and enable ongoing evaluation of discharge readiness.

It is recommended that assessing discharge readiness become a routine part of post-transplant care, covering both the patient’s clinical condition and psychosocial resources. This approach not only supports personalized care but also facilitates the implementation of interventions with measurable clinical outcomes, such as improved self-care knowledge, higher adherence, or fewer rehospitalizations. As a result, it enhances patient safety, boosts the quality of life for transplant recipients, and promotes the development of effective and sustainable healthcare policies.

## Figures and Tables

**Table 1:** Characteristics of the research group (N = 117).

	No.		%		
Sex					
Female	50		42.7		
Male	66		56.4		
Other	1		0.9		
Age (years)	M	Me	SD	Min	Max
	47.64	48.00	13.57	20.00	78.00
Education					
Primary	4		3.4		
Lower-secondary	0		0.0		
Vocational	17		14.5		
Secondary	53		45.3		
Tertiary	43		36.8		
Place of residence					
Country	48		41.0		
City of 20,000 to 50,000 inhabitants	20		17.1		
City of 51,000 to 100,000 inhabitants	9		7.7		
City of 101,000 to 500,000 inhabitants	9		7.7		
City of 201,000 to 500,000 inhabitants	6		5.1		
City of over 500,000 inhabitants	25		21.4		
Professional status					
Pupil/student	3		2.6		
Employed	66		56.4		
Unemployed	9		7.7		
Retired	20		17.1		
Occupational pension	19		16.2		
Duration of hospitalisation (days)	M	Me	SD	Min	Max
	19.62	16.00	11.49	5.00	74.00
Increased length of hospitalization due to complications					
Yes	41		35.0		
No	76		65.0		
Transplanted organ					
Kidney	80		68.4		
Liver	37		31.6		
Number of transplantation					
First	102		87.2		
Second	15		12.8		

M—mean; Me—median; SD—standard deviation; Min—minimum value; Max—maximum value.

**Table 2:** Educational activities.

	No.	%
Pre-Surgery Education		
Yes	89	76.1
No	28	23.9
Post-surgery education		
Yes	96	82.1
No	21	17.9
Educational materials received during hospitalization		
Yes	80	68.4
No	37	31.6
Independent undertaking of educational activities prior to surgery		
Yes	79	67.5
No	38	32.5

**Table 3:** Descriptive analysis of the C-HOBIC questionnaire.

Variables	M	Me	SD	A	K	Min	Max
Knowledge about medications	4.26	5.00	0.98	−1.37	1.33	1.00	5.00
Knowledge of the reasons for taking medications	4.46	5.00	0.85	−1.74	2.92	1.00	5.00
Ability to take prescribed medications	4.62	5.00	0.68	−2.35	7.55	1.00	5.00
Ability to notice health-related symptoms/changes	4.14	4.00	0.86	−0.77	0.32	1.00	5.00
Ability to follow treatment recommendations	4.54	5.00	0.69	−2.15	7.08	1.00	5.00
Knowledge related to persons to whom one can turn for help in performing daily activities	4.14	5.00	1.38	−1.56	1.31	0.00	5.00
Knowledge related to people/institutions to call in case of an emergency	4.15	5.00	1.11	−1.31	0.99	1.00	5.00
Ability to engage in daily physical activity	4.21	5.00	1.03	−1.45	1.70	1.00	5.00
Overall assessment of readiness for discharge	34.51	36.00	5.37	−1.42	2.90	11.00	40.00

M—mean; Me—median; SD—standard deviation; K—kurtosis; A—asymmetry; Min—minimum value; Max—maximum value.

**Table 4:** Descriptive analysis of self-care knowledge assessment, communication and information support and subjective assessment of readiness for discharge.

Variables	M	Me	SD	A	K	Min	Max
Subjective readiness	4.36	4.50	0.60	−1.02	1.79	1.75	5.00
Knowledge of self-care	4.39	4.83	0.82	−1.65	2.94	1.00	5.00
Communication and information support	4.53	5.00	0.75	−2.09	5.31	1.00	5.00

M—mean; Me—median; SD—standard deviation; K—kurtosis; A—asymmetry; Min—minimum value; Max—maximum value.

**Table 5:** Sociodemographic variables and assessment of readiness for discharge—C-HOBIC.

Variable/Scale	Sex			Age (Years)	Education				Place of Residence			Professional Status		
	F M ± SD	M* M ± SD	Z/*p*	Rho/*p*	Primary M ± SD	Secondary M ± SD	Tertiary M ± SD	H/*p*	Country M ± SD	City M ± SD	Z/*p*	Employed M ± SD	Unemployed M ± SD	Z/*p*
Overall assessment of readiness for discharge	4.25 ± 0.67	4.36 ± 0.67	−1.277/ 0.202	−0.065/ 0.485	4.27 ± 0.46	4.26 ± 0.85	4.40 ± 0.49	0.904/ 0.636	4.41 ± 0.67	4.25 ± 0.67	−1.553/ 0.120	4.38 ± 0.65	4.22 ± 0.70	−1.105/ 0.269
Knowledge about medications	4.02 ± 1.17	4.42 ± 0.79	−1.728/ 0.084	−0.075/ 0.422	4.19 ± 0.81	4.17 ± 1.07	4.40 ± 0.95	2.126/ 0.345	4.31 ± 1.07	4.22 ± 0.92	−1.080/ 0.280	4.30 ± 1.07	4.20 ± 0.87	−1.292 0.196
Knowledge of the reasons for taking medications	4.42 ± 0.88	4.48 ± 0.83	−0.302/ 0.762	−0.219/ 0.018	4.52 ± 0.51	4.34 ± 1.06	4.58 ± 0.66	0.769/ 0.681	4.54 ± 0.74	4.41 ± 0.91	−0.560/ 0.575	4.50 ± 0.88	4.41 ± 0.80	−1.234/ 0.217
Ability to take medication	4.58 ± 0.64	4.64 ± 0.72	−0.872/ 0.383	−0.126/ 0.175	4.52 ± 0.51	4.57 ± 0.84	4.72 ± 0.50	2.680/ 0.262	4.60 ± 0.68	4.62 ± 0.69	−0.151/ 0.880	4.72 ± 0.60	4.49 ± 0.76	−2.057/ 0.040
Ability to spot symptoms	4.14 ± 0.88	4.12 ± 0.85	−0.247/ 0.805	−0.084/ 0.368	4.19 ± 0.75	4.09 ± 0.99	4.16 ± 0.75	0.016/ 0.992	4.27 ± 0.87	4.04 ± 0.85	−1.666/ 0.096	4.20 ± 0.85	4.06 ± 0.88	−0.880/ 0.379
Ability to follow recommendations	4.54 ± 0.71	4.53 ± 0.68	−0.036/ 0.971	−0.189/ 0.041	4.52 ± 0.51	4.42 ± 0.86	4.70 ± 0.46	2.842/ 0.241	4.52 ± 0.80	4.55 ± 0.61	−0.256/ 0.798	4.59 ± 0.72	4.47 ± 0.64	−1.463/ 0.143
Knowledge of assistance with daily activities	4.12 ± 1.27	4.14± 1.47	−0.450/ 0.653	−0.104/ 0.266	3.81 ± 1.54	4.13 ± 1.39	4.30 ± 1.28	2.326/ 0.313	4.35 ± 1.08	3.99 ± 1.54	−0.944/ 0.345	4.41 ± 1.14	3.78 ± 1.58	−2.134/ 0.033
Knowledge of institutions in the event of an emergency	4.08 ± 1.07	4.20± 1.15	−1.038/ 0.299	−0.026/ 0.778	4.29 ± 0.90	4.21 ± 1.18	4.02 ± 1.12	1.564/ 0.457	4.40 ± 0.89	3.99 ± 1.22	−1.702/ 0.089	4.15 ± 1.18	4.16 ± 1.03	−0.369/ 0.712
Ability to engage in physical activity	4.08 ± 1.14	4.33 ± 0.93	−1.203/ 0.229	0.073/ 0.433	4.14 ± 0.79	4.19 ± 1.09	4.28 ± 1.08	1.525/ 0.467	4.29 ± 0.94	4.16 ± 1.09	−0.409/ 0.683	4.21 ± 1.09	4.22 ± 0.97	−0.318/ 0.750

F—female, M*—male, M—mean; SD—standard deviation; Rho—Spearman coefficient; H—Kruskal–Wallis test; Z—Mann–Whitney test.

**Table 6:** Clinical variables and assessment of readiness for discharge (C-HOBIC).

Variable/Scale	Length of Stay in Hospital	Transplanted Organ			Transplantation			Extension Of Stay Due to Complications		
	(Days) Rho/*p*	Kidney M ± SD	Liver M ± SD	Z/*p*	First M ± SD	Second M ± SD	Z/*p*	Yes M ± SD	No M ± SD	Z/*p*
Overall assessment of readiness for discharge	−0.044/0.638	4.33 ± 0.69	4.29 ± 0.64	−0.333/0.739	4.27 ± 0.70	4.61 ± 0.33	−1.536/0.125	4.30 ± 0.68	4.32 ± 0.67	−0.241/0.809
Knowledge about medications	−0.147/0.113	4.24 ± 1.03	4.30 ± 0.88	−0.023/0.982	4.23 ± 0.99	4.47 ± 0.92	−1.050/0.294	4.22 ± 0.99	4.28 ± 0.99	−0.468/0.639
Knowledge about the reasons for taking medications	−0.083/0.376	4.50 ± 0.86	4.38 ± 0.83	−1.021/0.307	4.42 ± 0.88	4.73 ± 0.46	−1.111/0.267	4.44 ± 0.90	4.47 ± 0.82	−0.227/0.820
Ability to take prescribed medications	0.041/0.658	4.58 ± 0.74	4.70 ± 0.52	−0.683/0.495	4.58 ± 0.71	4.87 ± 0.35	−1.606/0.108	4.68 ± 0.72	4.58 ± 0.66	−1.150/0.250
Ability to spot symptoms/health changes	−0.094/0.316	4.16 ± 0.88	4.08 ± 0.83	−0.679/0.497	4.08 ± 0.88	4.53 ± 0.64	−1.928/0.054	4.02 ± 0.85	4.20 ± 0.86	−1.190/0.234
Ability to comply with therapeutic recommendations	−0.033/0.721	4.46 ± 0.76	4.70 ± 0.46	−1.574/0.115	4.52 ± 0.71	4.67 ± 0.49	−0.596/0.551	4.51 ± 0.64	4.55 ± 0.72	−0.648/0.517
Knowledge of people supporting in daily activities	0.002/0.984	4.18 ± 1.39	4.05 ± 1.37	−0.652/0.514	4.05 ± 1.44	4.73 ± 0.46	−1.417/0.157	4.17 ± 1.46	4.12 ± 1.34	−0.318/0.751
Knowledge of persons/institutions to contact in an emergency	−0.067/0.474	4.26 ± 1.04	3.92 ± 1.23	−1.423/0.155	4.06 ± 1.15	4.80 ± 0.41	−2.535/0.011	4.12 ± 1.14	4.17 ± 1.10	−0.131/0.896
Ability to engage in daily physical activity	0.101/0.280	4.23 ± 0.98	4.19 ± 1.15	−0.272/0.785	4.24 ± 1.03	4.07 ± 1.10	−0.593/0.553	4.22 ± 1.13	4.21 ± 0.98	−0.478/0.633

M—mean; SD—standard deviation; Rho—Spearman coefficient; Z—Mann–Whitney test.

**Table 7:** Correlation between overall readiness for discharge, subjective readiness for discharge, self-care knowledge, communication and information support and psychological resilience, social support, levels of depressive symptoms, and anxiety.

	Overall Assessment of Readiness for Discharge		Subjective Readiness		Knowledge of Self-Care		Communication and Information Support	
	Rho	*p*	Rho	*p*	Rho	*p*	Rho	*p*
SPP-25								
Perseverance and determination in action	0.253	0.006	−0.005	0.955	0.179	0.053	0.169	0.069
Openness to new experiences and a sense of humor	0.345	<0.001	0.111	0.232	0.212	0.022	0.261	0.004
Personal coping skills and tolerance of negative emotions	0.338	<0.001	0.128	0.171	0.238	0.010	0.261	0.004
Tolerance for failure and treating life as a challenge	0.406	<0.001	0.110	0.237	0.306	0.001	0.270	0.003
Optimistic attitude to life and ability to mobilize in difficult situations	0.428	<0.001	0.097	0.299	0.291	0.001	0.345	<0.001
SPP-25 overall score	0.389	<0.001	0.096	0.303	0.271	0.003	0.291	0.001
MSPSS								
Friends	0.147	0.113	0.159	0.086	0.082	0.381	0.331	<0.001
Family	0.131	0.159	0.200	0.030	0.153	0.099	0.291	0.001
Significant others	0.166	0.073	0.182	0.049	0.161	0.083	0.229	0.013
MSPSS overall score	0.144	0.122	0.202	0.029	0.126	0.176	0.313	0.001
HADS								
Depression	−0.204	0.028	−0.102	0.276	−0.119	0.200	−0.194	0.036
Anxiety	−0.182	0.050	−0.193	0.037	−0.214	0.021	−0.317	<0.001

Rho—Spearman’s rho coefficient, SPP-25—Psychological Resilience Scale, MSPSS—Multidimensional Scale of Perceived Social Support, HADS—Hospital Anxiety and Depression Scale.

**Table 8:** Correlation between subjective readiness, communication and information support, knowledge of self-care and discharge readiness and its aspects.

	Subjective Readiness		Communication and Information Support		Knowledge of Self-Care	
	Rho	*p*	Rho	*p*	Rho	*p*
Overall assessment of readiness for discharge	0.416	<0.001	0.611	<0.001	0.660	<0.001
Knowledge about medications	0.282	0.002	0.532	<0.001	0.493	<0.001
Knowledge of the reasons for taking medications	0.335	<0.001	0.529	<0.001	0.608	<0.001
Ability to take prescribed medications	0.399	<0.001	0.479	<0.001	0.499	<0.001
Ability to notice health-related symptoms/changes	0.319	<0.001	0.399	<0.001	0.582	<0.001
Ability to follow treatment recommendations	0.361	<0.001	0.339	<0.001	0.402	<0.001
Knowledge related to persons to whom one can turn for help in performing daily activities	0.427	<0.001	0.445	<0.001	0.475	<0.001
Knowledge related to people/institutions to call in case of an emergency	0.208	0.024	0.435	<0.001	0.490	<0.001
Ability to engage in daily physical activity	0.236	0.010	0.366	<0.001	0.312	0.001

Rho—Spearman’s rho coefficient.

**Table 9:** Regression model with analyzed predictors.

	Non-Standardized Coefficients		Standardized Coefficients	t	p	VIF
	B	Standard Error	Beta			
(Constant)	0.996	0.614		1.622	0.108	
SPP-25	0.011	0.005	0.221	2.381	0.019	1.727
HADS-depression	0.005	0.023	0.023	0.197	0.844	2.763
HADS-anxiety	−0.001	0.031	−0.002	−0.020	0.984	2.338
Friends	0.005	0.011	0.047	0.494	0.622	1.803
Family	0.001	0.020	0.003	0.026	0.980	2.737
Significant person	0.003	0.019	0.017	0.162	0.872	2.115
Knowledge of self-care	0.475	0.088	0.577	5.424	<0.001	2.275
Communication and information support	0.023	0.097	0.025	0.236	0.814	2.314

F = 11.629; *p* < 0.001; Adjusted R-square = 0.423.

## Data Availability

The data presented in this study are available on request from the corresponding author due to ethical and legal restrictions related to data confidentiality. The data are recorded in the researchers’ native language.
